# Alda-1 Attenuates Hyperoxia-Induced Acute Lung Injury in Mice

**DOI:** 10.3389/fphar.2020.597942

**Published:** 2021-01-08

**Authors:** Sahebgowda Sidramagowda Patil, Helena Hernández-Cuervo, Jutaro Fukumoto, Sudarshan Krishnamurthy, Muling Lin, Matthew Alleyn, Mason Breitzig, Venkata Ramireddy Narala, Ramani Soundararajan, Richard F. Lockey, Narasaiah Kolliputi, Lakshmi Galam

**Affiliations:** ^1^ Division of Allergy and Immunology, Department of Internal Medicine, Morsani College of Medicine, University of South Florida, Tampa, FL, United States; ^2^ Department of Molecular Medicine, Morsani College of Medicine, University of South Florida, Tampa, FL, United States; ^3^ Brown School, Washington University, St. Louis, MO, United States; ^4^ Department of Zoology, Yogi Vemana University, Kadapa, India

**Keywords:** Oxidative Stress, Hyperoxia, acute lung injury, Alda-1, aldehyde dehydrogenase 2, HALI

## Abstract

Acute lung injury (ALI), a milder form of acute respiratory distress syndrome (ARDS), is a leading cause of mortality in older adults with an increasing prevalence. Oxygen therapy, is a common treatment for ALI, involving exposure to a high concentration of oxygen. Unfortunately, hyperoxia induces the formation of reactive oxygen species which can cause an increase in 4-HNE (4-hydroxy 2 nonenal), a toxic byproduct of lipid peroxidation. Mitochondrial aldehyde dehydrogenase 2 (ALDH2) serves as an endogenous shield against oxidative stress-mediated damage by clearing 4-HNE. Alda-1 [(N-(1, 3 benzodioxol-5-ylmethyl)-2, 6- dichloro-benzamide)], a small molecular activator of ALDH2, protects against reactive oxygen species-mediated oxidative stress by promoting ALDH2 activity. As a result, Alda-1 shields against ischemic reperfusion injury, heart failure, stroke, and myocardial infarction. However, the mechanisms of Alda-1 in hyperoxia-induced ALI remains unclear. C57BL/6 mice implanted with Alzet pumps received Alda-1 in a sustained fashion while being exposed to hyperoxia for 48 h. The mice displayed suppressed immune cell infiltration, decreased protein leakage and alveolar permeability compared to controls. Mechanistic analysis shows that mice pretreated with Alda-1 also experience decreased oxidative stress and enhanced levels of p-Akt and mTOR pathway associated proteins. These results show that continuous delivery of Alda-1 protects against hyperoxia-induced lung injury in mice.

## Introduction

There are more than 200,000 annual cases of acute lung injury (ALI) in the United States with a mortality rate of 40–50% (Rubenfeld et al., 2005; Galam et al., 2015). ALI patients commonly use oxygen therapy as a treatment; however, high concentrations of oxygen can exacerbate the condition and cause further alveolar injury (Galam et al., 2015). Hyperoxia induces ALI in small animal and primate models (Fukumoto et al., 2013; Kallet and Matthay, 2013). It promotes inflammation and causes infiltration of cytokines, macrophages, and neutrophils, and results in edema, alveolar damage, and death (Fukumoto et al., 2013; Kallet and Matthay, 2013; Fukumoto et al., 2016; Narala et al., 2018). Therefore, hyperoxia is a relevant model to study the pathophysiology of lung injury and to explore the molecular mechanisms of ALI.

ALDH2, an enzyme expressed in liver and lungs, serves as an invaluable shield against oxidative stress-mediated damage and detoxifies reactive aldehydes, such as 4-hydroxy-2-nonenal (4-HNE) (Chen et al., 2014; Breitzig et al., 2016; Patil et al., 2019). There also exists a naturally occurring mutant version of ALDH2, termed ALDH2*2, with reduced levels of ALDH2 activity. In humans, this mutation is associated with increased susceptibility to chronic obstructive pulmonary disease (COPD), tuberculosis, and asthma (Park et al., 2014; Tu et al., 2014). Human carriers of ALDH2*2 (mutant) show evidence of reduced lung function (Kuroda et al., 2017).

Alda-1, an ALDH2 activator, enhances ALDH2 activity by binding near its Glu268 and Cys302 residues (Beretta et al., 2010). The resultant activity of ALDH2 is able to protect against ischemic reperfusion injury, heart failure, stroke, and myocardial infarction in animal models. Alda-1 blunts oxidative stress-mediated damage of various human cell types, including cardiac, lung endothelial, brain endothelial, umbilical vein endothelial, and hepatic cells. It also reduces damage done to the lung, liver, brain, intestine, kidney, and eye in animal models (Perez-Miller et al., 2010; Chen et al., 2014; Fu et al., 2014; Lu et al., 2017; Zhu et al., 2017; Zhang et al., 2018; Patil et al., 2019). Bolstering ALDH2 activity via Alda-1 also inhibits immune cell infiltration, bronchoalveolar protein leakage, alveolar damage, oxidative stress responses during severe hemorrhagic shock, and acrolein-induced ALI in animal models (Lu et al., 2017; Hua et al., 2019). Conversely, exposure to hyperoxia elevates the concentrations of ROS induced 4-HNE and promotes immune cell infiltration, alveolar damage, and enhanced oxidative stress (Chen et al., 2010; Fukumoto et al., 2013; Cox et al., 2015; Galam et al., 2015; Breitzig et al., 2016; Wedgwood et al., 2016; Fukumoto et al., 2019). However, it is unknown whether hyperoxia-induced oxidative stress can be mitigated by Alda-1 pretreatment. Therefore, this study evaluates the potential therapeutic effects of Alda-1 in HALI in mice.

## Materials and Methods

### Chemicals/Reagents

Alda-1, PEG (polyethylene glycol) (Catalog: 90878-1L-F) and DMSO (dimethyl sulfoxide) (catalog: D2650-100ML) (Sigma Aldrich, St. Louis, MO), were used in this work.

### Mice

The institutional animal care and use committee (IACUC) of USF approved all animal experimental procedures. The C57BL/6 were obtained from Envigo (Indianapolis, IN). C57BL/6 mice were maintained in comparative medicine (COM) animal facility under similar conditions of a 12-h dark-12-h light cycle, humidity (60 ± 5%), and temperature (22 ± 1°C). All mice used in the study were ages 7–9 wk old and mice to study ALI and ARDS based on earlier established studies (Lu et al., 2017; He et al., 2005; O'Reilly et al., 2000; Reddy et al., 2011; Sauler et al., 2015; Zhang et al., 2013) and received a regular diet *ad libitum*.

### *In vivo* Hyperoxia

C57BL/6 mice (*n* = 4) were exposed to 100% oxygen (hyperoxia) for 48 h in airtight cages (75 × 50 × 50 cm). A proOx p100 sensor (Biospherix, New York, NY) was used to measure oxygen concentrations in the chamber as described previously (Kolliputi and Waxman, 2009; Waxman and Kolliputi, 2009).

### Surgical Implantation of Alzet Pumps

The mice implanted with Alzet pumps were divided into two groups under hyperoxia. One was vehicle/control [50% DMSO: 50% PEG] and other was Alda-1 pretreatment (8 mg/kg/h) [50% Alda-1: 50% PEG]. 200 µl of control and Alda-1 was added into each Alzet pump and immersed under sterile PBS for 3 h at 37°C under sterile conditions for priming according to manufacturer instructions (Sigma Aldrich). After priming, the anesthesia was given to the mice and Alzet pump were inserted on dorsal side 24 h prior to hyperoxia exposure. The mice cages were kept on heat pad after insertion of Alzet pumps. Subcutaneously implanted Alzet pumps (Cupertino, CA) were used to continuously administer either the vehicle or Alda-1 for 48 h to mice under hyperoxia exposure as previously reported (Sun et al., 2011). The final concentration of Alda-1 was 20 µM because at this concentration Alda-1 inhibits 4-HNE induced Aldh2 inactivation (Perez-Miller et al., 2010). After 48 h of hyperoxia, the mice were anesthetized with ketamine/xylazene and after cervical dislocation of mice, following perfusion, lungs were collected (Supplementary Figure 1).

### Bronchoalveolar Lavage Fluid Analysis

BAL (Bronchoalveolar lavage) fluid was collected after 48 h of hyperoxia exposure. Total cell counts of BAL fluid were performed using an hemocytometer and cytospin fraction subjected to a differential cell count by Diff-Quik (Thermo Fisher, Waltham, MA); counts were followed by imaging as described previously (Fukumoto et al., 2013; Galam et al., 2015). The protein concentration in BAL fluid was measured by utilizing a BCA protein assay kit (Thermo Fisher, Waltham, MA).

### Lung Histology

After euthanizing the mice, lungs were perfused in saline and the left lung was fixed in 4% paraformaldehyde (4% PFA, Electron Microscopy Sciences, Hatfield, PA) for 48 h, embedded in paraffin and subjected to Hematoxylin and Eosin (H&E) staining followed by imaging as described previously (Fukumoto et al., 2013).

### Evaluation of Lung Injury

Analysis of H&E-stained tissue sections determined the degree of lung injury and pathological severity using previously described scoring criteria (Sue et al., 2004; Jain et al., 2008). The parameters for evaluation of lung injury were alveolar congestion, immune cell infiltration, and interstitial thickening of alveoli. The categories of scoring were as follows: 0 for normal lung with no abnormalities; 1 for wounds involving <25%; 2 for wounds involving 25–50%; 3 for wounds involving 50–75%; 4 for wounds involving >75% of the lung. The overall histopathological scoring was averaged and plotted.

### Western Blotting

Three groups of mice each received one of the following exposures: normoxia, hyperoxia, or hyperoxia + Alda-1. The mice were euthanized, perfused with saline, and lung samples were collected, and flash frozen in liquid nitrogen. Lung samples were then pulverized, homogenized in lysis buffer (20 mM Tris-HCl, pH 7.4, 150 mM NaCl, and 0.5% Triton X-100), and centrifuged at 21,000 *g* for 15 min at 4°C to obtain the soluble protein extract. The protein concentrations were estimated by BCA assay, and equal amounts of protein were loaded (15 µg) on a SDS-PAGE gel and subjected to immunoblot analysis. The primary antibodies cytochrome c, Akt, p-Akt (Ser 473), mammalian target of rapamycin (mTOR), p-mTOR (Ser 2448), phospho-p70 s60 kinase (ser 371) and phospho-p70 s60 kinase (Thr 389) (cell signaling technology, Danvers, MA) were used. The secondary antibodies were goat anti-rabbit HRP conjugated (Jackson Immunoresearch, West grove, PA). KwikQuant ECL solution (Kindle Biosciences, Greenwich, CT) was used to visualize proteins. β-actin was used as a loading control. Protein bands were quantitated using Image J (NIH, Bethesda, MD) after normalizing to β-actin using Image J software.

### Statistical Analysis

Student’s t-test was used to determine statistical significance. The data are expressed as Mean ± S.E.M. *p* < 0.05 was considered the threshold for significance.

## Results

### Pretreatment of Alda-1 Attenuates Hyperoxia-Triggered Immune Cell Infiltration

**FIGURE 1:**
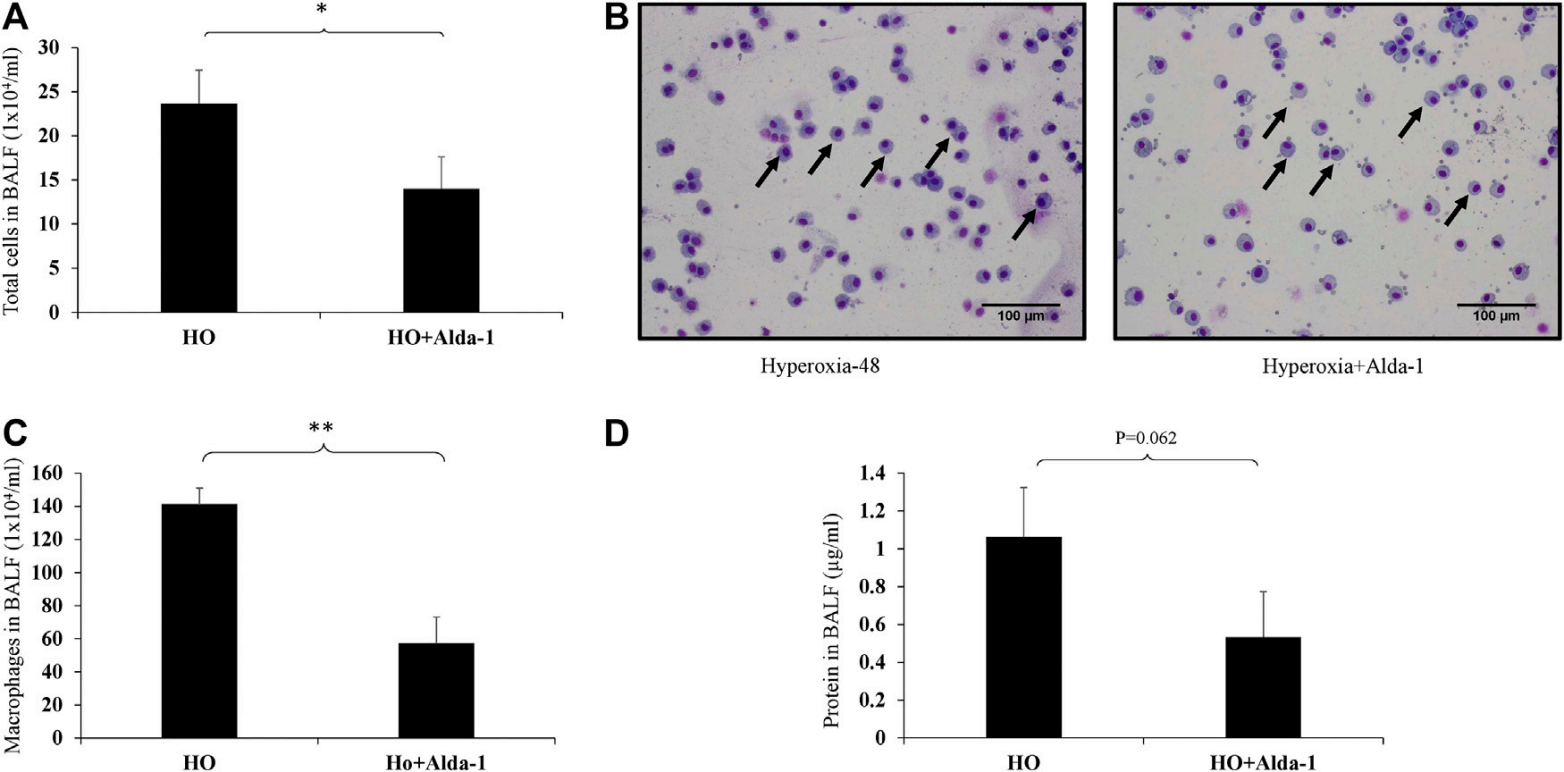
Alda-1 suppresses hyperoxia-induced infiltration of immune cells infiltration into alveolar space: C57BL/6 mice were divided into three groups: normoxia, hyperoxia, or hyperoxia + Alda-1. Mice were pretreated with Alda-1 or vehicle for 48 h and then dissected. BAL fluid was collected and subjected to cell counts using a hemocytometer or cytospin followed by Diff- Quik staining. **(A)** Microscopic evaluation of cell totals following cytospin of BAL fluid collected from normoxia, hyperoxia, and hyperoxia + Alda-1 mice. **(B)** Diff-Quik staining from normoxia, hyperoxia, and hyperoxia + Alda-1 treated mice (arrow indicates macrophages) (magnification = ×200). **(C)** Microscopic evaluation of macrophage number in BAL fluid collected from normoxia, hyperoxia, and hyperoxia + Alda-1 mice. **(D)** Analysis of total BAL fluid protein by BCA assay; representative images are shown from at least three mice/group (*p < 0.05, **p < 0.005).

Hyperoxia exposure causes an increase in immune cell infiltration in the lung airspaces (Rubenfeld et al., 2005; Nagato et al., 2015). Analysis of BAL fluid is an indicator of immune cell infiltration and inflammation in lungs (Narala et al., 2018). BAL fluid analysis was performed to assess the total number of immune cells and the types of immune cells present were assessed using differential cell counts. Alda-1 pretreated mice exposed to hyperoxia showed a significant decrease (1.7- fold) in total cell counts vs. the hyperoxia control group (Figure 1A). There was a significant increase in alveolar macrophages in mice exposed to 48 h of hyperoxia (Figures 1B,C). The total protein in the BAL fluid of Alda-1 pretreated mice exposed to hyperoxia showed a 2-fold decrease relative to the hyperoxia control that was not statistically significant, (*p* = 0.062) (Figure 1D).

### Pretreatment of Alda-1 attenuates hyperoxia-induced alveolar damage and lung inflammation

**FIGURE 2:**
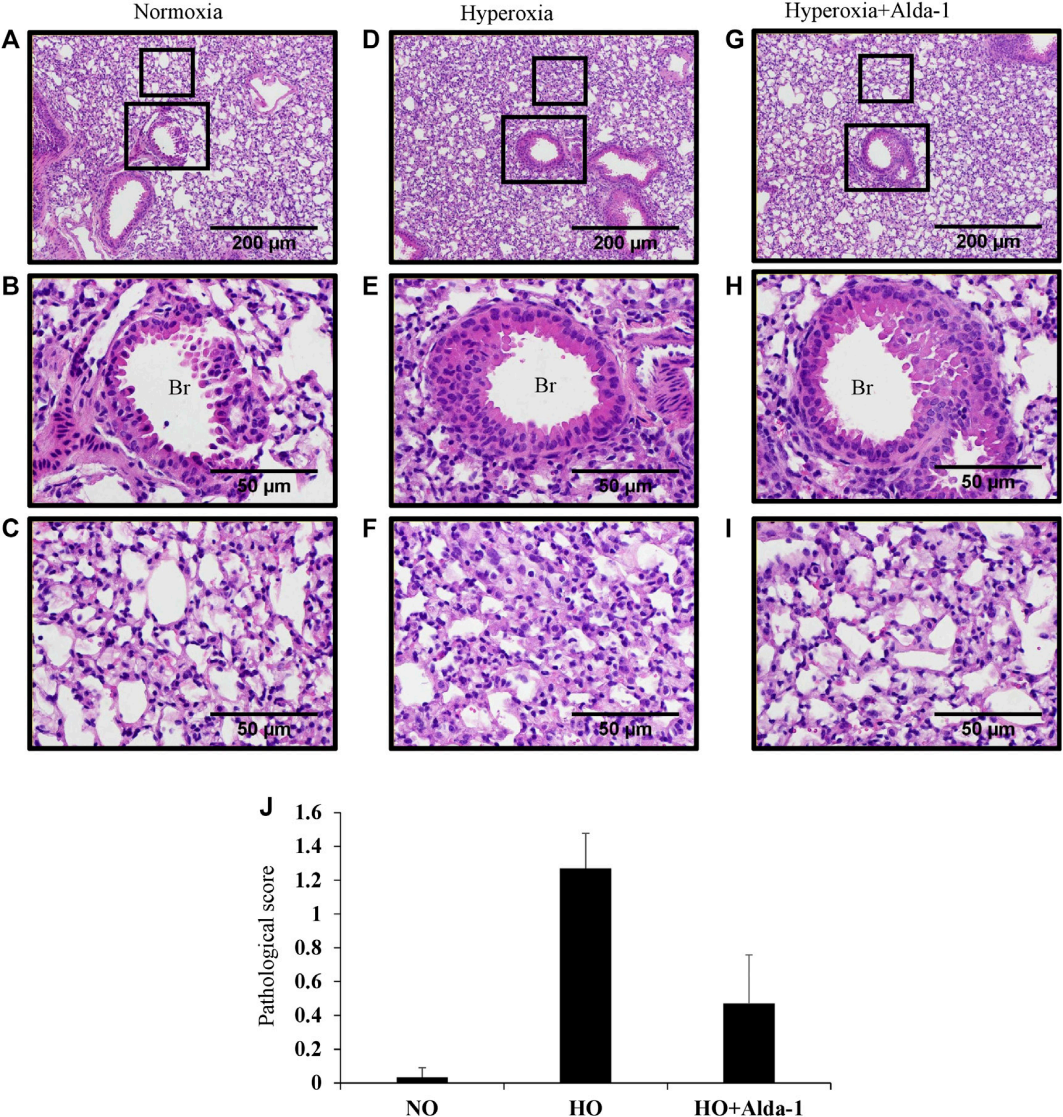
Alda-1 suppresses hyperoxia induced alveolar damage and lung inflammation: C57BL/6 mice were divided into three groups: normoxia, hyperoxia, or hyperoxia + Alda-1. Representative photomicrographs of the H&E stained lungs derived from the three groups. Br: Bronchiolar area. Magnification: ×100 and ×400 and scale bar: 200 and 50 µm **(A–I)**. Histological changes were given a pathological score **(J)**. Representative H&E images from at least three mice/group. Data are shown as means with SE or SD.

Hyperoxia causes thickening of bronchial epithelium, alterations in alveolar permeability, and lung inflammation (Cox et al., 2015; Narala et al., 2018; Fukumoto et al., 2019). Mice were exposed to 48 h of hyperoxia with or without Alda-1 pretreatment. H&E staining of lung samples reveals that mice pretreated with Alda-1 during hyperoxia have no difference in bronchial epithelium thickness vs. those treated without Alda-1 during hyperoxia (Figures 2D,G). The inset shows bronchioles (Figures 2E,H). However, analysis shows increased alveolar damage in the hyperoxia group (Figure 2F) compared to the normoxia group (Figure 2C) and mice that received hyperoxia exposure with Alda-1 (Figure 2I) pretreatment. The pathological features of inflammation were assessed by measuring alveolar congestion, immune cell infiltration, and thickening of alveolar interstitium. The H&E stained images were also assigned scores. Hyperoxia increased the pathological score by 38-fold relative to the normoxia control, whereas pretreatment with Alda-1 reduced the score by three-fold (Figure 2J). After hyperoxia, some of the mice were found to have a hunched posture.

### Pretreatment of Alda-1 Attenuates Oxidative Stress During Hyperoxia Exposure

**FIGURE 3:**
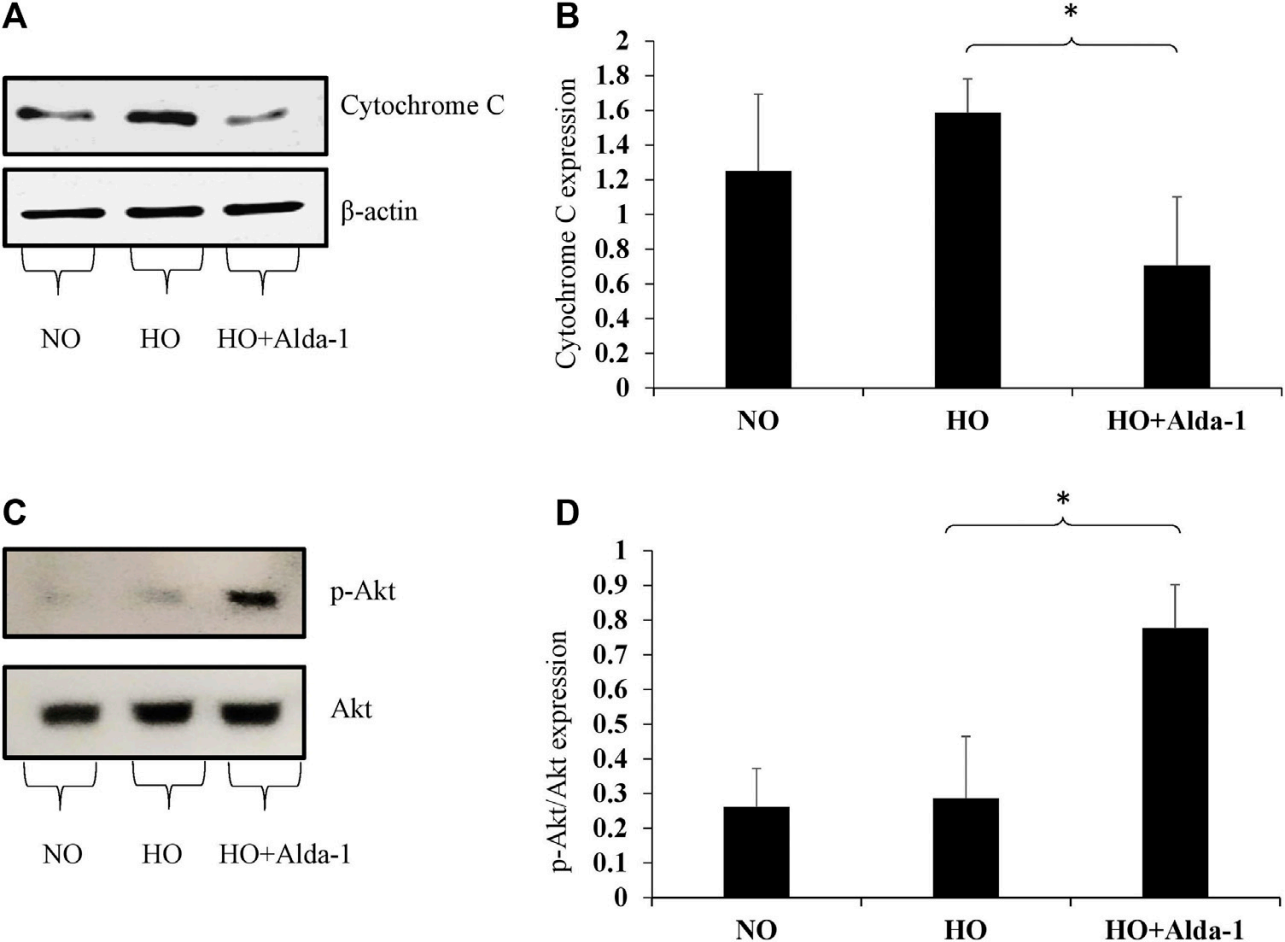
Alda-1 suppresses oxidative stress: Three groups of C57BL/6 mice received exposure to normoxia, hyperoxia, or hyperoxia + Alda-1 for 48 h. Whole cell lysate collected from lung samples was subjected to western blot analysis for cytochrome c **(A)** and *p*-Akt **(C)**; β-actin and Akt were used as loading controls, respectively. Densitometry analysis shows the expression of cytochrome c **(B)** and *p*-Akt **(D)**. Representative blots from at least three mice/group, (*p < 0.05, **p < 0.005).

Western blot analysis was carried out using the lung lysates from C57BL6 mice exposed to 48 h of hyperoxia, with or without Alda-1 pretreatment, to assess the markers of oxidative stress. Hyperoxia causes oxidative stress and affects cell survival (Eklow-Lastbom et al., 1986; Jenkins et al., 2018; Patil et al., 2019). The marker of oxidative stress, cytochrome c, was assessed by western blotting. Hyperoxia increased the protein levels of cytochrome c by 1.26-fold relative to the normoxia control group (Figures 3A,B). Alda-1 pre-treatment prior to hyperoxia, vs. hyperoxia without Alda-1 pretreatment, significantly decreased the levels of cytochrome c by 2.25-fold.

Akt/p-Akt (ser473) plays a key role in cell proliferation and cell growth and also attenuates the oxidative stress caused by elevation of ROS (Chua et al., 2009). The p-Akt (ser 473) is the sole marker for Akt activation that is independent of the less enzymatically reactive Thr 308 isoform (Vincent et al., 2011). Western blot analysis revealed that total Akt expression did not change between the three groups. However, the p-Akt/total Akt ratio significantly increased by 2.71-fold in the Alda-1 pretreatment group relative to hyperoxia or normoxia controls (Figures 3C,D). These results indicate that Alda-1 pretreatment during hyperoxia not only decreases oxidative stress, but also enhances cell survival.

### Pretreatment of Alda-1 activates survival during hyperoxia via the mechanistic target of rapamycin kinase (mTOR) pathway

mTOR kinase is a regulator of cell growth, proliferation, and survival (Smith et al., 2014; Wedgwood et al., 2016). The mTOR pathway is conserved (Schalm and Blenis, 2002) and composed of two complexes: mTORC1 and mTORC2 and their catalytic subunit mTOR (Zhou and Huang, 2010). mTOR activation is necessary for cellular growth and development (Smith et al., 2014; Wedgwood et al., 2016). Proteins, such as mTOR, p-mTOR (ser 2448), and phospho p-70 S6 kinases, play an important role in protein translation and cell survival (Chiang and Abraham, 2005; Chen et al., 2010; Smith et al., 2014). The proteins of the mTOR pathway were analyzed by western blot analysis.

**FIGURE 4:**
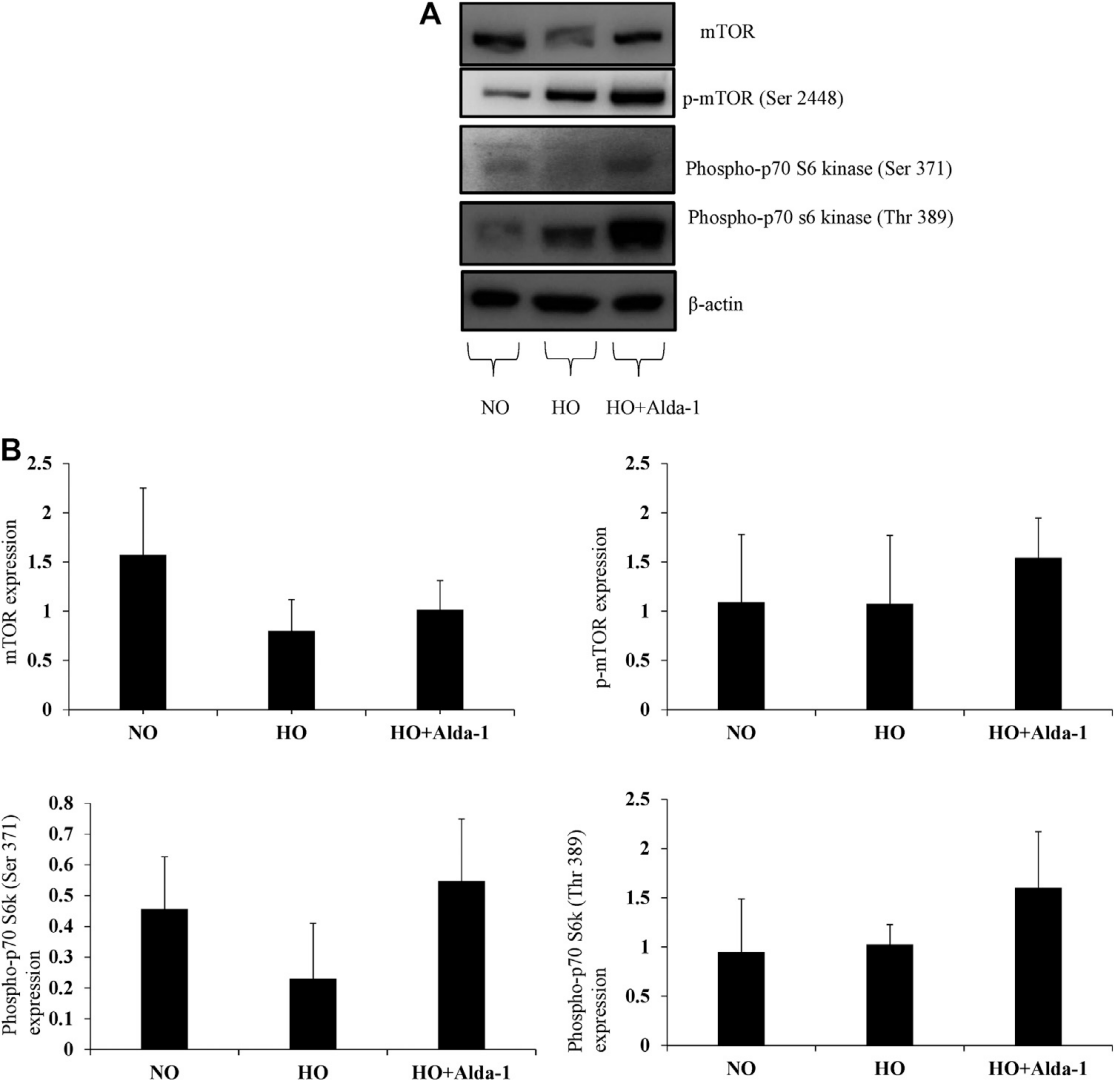
Alda-1 attenuates hyperoxic lung injury via mTOR pathway: Three groups of C57BL/6 mice received exposure to normoxia, hyperoxia, or hyperoxia + Alda-1 for 48 h. Whole cell lysate was collected from lung samples and subjected to western blot analysis for mTOR, *p*-mTOR (ser 2448), phospho-p70 s60 kinase (ser 371), and phospho-p70 s60 kinase (Thr 389) **(A)**. β-actin was used as a loading control. Densitometry analysis shows the expression of mTOR, *p*-mTOR (ser 2448), phospho-p70 s60 kinase (ser 371), phospho-p70 s60 kinase (Thr 389) **(B)**. Representative blots from at least three mice/group.

The data show a decrease in total mTOR by two-fold in the hyperoxia group relative to the normoxia control (Figures 4A,B). Pretreatment with Alda-1, followed by hyperoxia, caused an increase in mTOR protein levels by 1.27-fold, p-mTOR (Ser 2448) by 1.43-fold, phosphor p-70 s6 kinase (Ser 371) by 2.3 fold, and phosphor p-70 s6 kinase (Thr 389) by 1.56-fold, respectively versus hyperoxia without Alda-1 pretreatment (Figures 4A,B). However, the results were not statistically significant.

## Discussion

This study evaluated the protective role of Alda-1 in HALI murine model for the first time. Pretreatment with Alda-1 prior to exposing mice to hyperoxia suppresses infiltration of immune cells, alveolar damage, lung inflammation, and oxidative stress and enhances cell survival via the Akt and mTOR pathways. In other studies Alda-1 was administered intraperitoneally (Zhu et al., 2017; Hua et al., 2019)and in this study Alda-1 administration was via Alzet pumps in a sustained fashion due to the short half-life and this pretreatment was continued throughout the hyperoxia exposure for 48 h (Zambelli et al., 2014).

Hyperoxia is known to increase ROS levels (Galam et al., 2015; Breitzig et al., 2016). The oxidative stress induced by ROS is implicated in the pathogenesis of several diseases including ALI (Galam et al., 2016). 4-HNE, a byproduct of lipid peroxidation, increases in HALI and forms protein adducts with lysine, histidine, and cysteine residues causing cell death (Waxman and Kolliputi, 2009; Galam et al., 2015). Furthermore, hyperoxia prompts the release of pro-apoptotic signals by damaging nearby cells (Pagano et al., 2004), and triggers infiltration of immune cells. These events lead to edema, alveolar and capillary protein leakage, and perfusion of blood plasma into alveoli (Mach et al., 2011; Narala et al., 2018). ALDH2 plays a key role in protection against HALI (Chen et al., 2014). It is an endogenous enzyme that is involved in clearing 4-HNE and is required for lung homeostasis. Alda-1, a potent activator of ALDH2, protects against oxidative stress in models of ischemia by bolstering the activity of ALDH2 (Solito et al., 2013; Fu et al., 2014; Zhang et al., 2018). In 2019 we published a paper on the use of Alda-1 *in vitro*, where Alda-1 pretreatment shielded lung endothelial cells from HALI by decreasing 4-HNE levels (Patil et al., 2019).

This study shows that pretreatment of mice with Alda-1 in a sustained fashion, using Alzet pumps, decreases protein leakage and immune cell infiltration. The alveolar capillary network plays a critical role in the control of immune cell infiltration, but hyperoxia readily damages this barrier (Lagishetty et al., 2014; Cox et al., 2015). The ability of Alda-1 during hyperoxic conditions to suppress infiltration of immune cells into BAL fluid may preserve the integrity of the alveolar barrier, as demonstrated in this study. Histopathological analysis of Alda-1 pretreated mice exposed to hyperoxia further supports this observation indicating a preservation of alveolar capillary barrier integrity when compared to hyperoxia alone. This finding is consistent with decreased macrophage infiltration and BAL protein analysis in the Alda-1 pretreated group. These results suggest that Alda-1 suppresses alveolar damage and lung inflammation during hyperoxia. Several reports show Alda-1 is able to attenuate liver inflammation, hemorrhagic-shock-induced lung injury, and intestinal injury (Zhu et al., 2017; Hua et al., 2019).

Western blot analysis was carried out to dissect the signaling pathways implicated in Alda-1 mediated protection against HALI. Hyperoxia causes a release of cytochrome c from the mitochondrial membrane into the cytosol and subsequently activates caspase induced apoptosis (Pagano et al., 2004). Hyperoxia exposure causes mitochondrial dysfunction, enhanced mitochondrial ROS by altering the expression of mitochondrial proteins like Pink1, Parkin, Mfn1, Mfn2, OPA1, DRP1 (Zhang et al., 2014). Hyperoxia exposure affects mitochondrial membrane potential (Solito et al., 2013). The use of Alda-1 balances the kinetic properties of mitochondrial Aldh2 (Belmont-Diaz et al., 2016) and enhances mitochondrial membrane potential and inhibits mitochondrial ROS production (Solito et al., 2013; Chiu et al., 2015). Alda-1 pretreatment enhanced the mitochondrial membrane potential in endothelial cells of lung, brain, umbilical vein cells and inhibited ROS production in neuronal cells (Solito et al., 2013; Chiu et al., 2015; Patil et al., 2019).

Prolonged hyperoxia exposure causes decrease in p-Akt expression (Alphonse et al., 2011; Wu et al., 2018). This phenomenon also occurs in neurodegenerative diseases, heart failure, and traumatic brain injury in humans (Pagano et al., 2004). Akt plays an important role in regulation of signaling pathways involved in cell proliferation, survival, and metabolism, whereas hyperoxia suppresses the phosphorylation of Akt by PI3K (Wu et al., 2018). We hypothesized that Alda-1 pretreatment would decrease cytochrome c and increase the expression of p-Akt. Supporting our hypothesis, the results indicate that pretreatment of Alda-1 prior to hyperoxia significantly decreases cytochrome c levels and enhances p-Akt expression. The p-Akt levels are slightly increased in hyperoxia vs. normoxia in our results because the cells have antioxidant mechanism and try to alleviate the harmful molecules with numerous defense mechanisms (Jamieson, 1998; Outten et al., 2005). The defense mechanism includes thioredoxin, glutathione and methionine sulfate reductase against oxidative stress (Jamieson, 1998; Outten et al., 2005). Cell survival via Akt pathway is implicated in lung epithelial cell during HALI and the Akt activation during normoxia is retained during hyperoxia (Xu et al., 2006). This corroborates the findings of a previous study that demonstrated the importance of the PI3K/Akt pathway in hyperoxic lung injury (Kolliputi and Waxman, 2009). Administration of Alda-1 has also been shown to reduce cytochrome c expression in endothelial cells from the lungs, umbilical veins, and brain (Solito et al., 2013; Patil et al., 2019) and possibly Alda-1 accelerated Akt signaling by inhibiting toxic aldehyde. However, more work needs to be done to know how Alda-1 activates Akt signaling in HALI.

The mTOR pathway was evaluated to understand the effect of Alda-1 downstream of Akt signaling. Its deletion is harmful as mTOR-knockout mice show heart failure, abnormal cell cycle progression in neurons, and inhibition of embryonic stem cell development (Yan et al., 2000; Zhang et al., 2010; Vadysirisack and Ellisen, 2012). Hyperoxia alters mTOR subscrates and inhibits protein synthesis (Konsavage et al., 2010). mTOR auto phosphorylates in response to hormone or growth factor stimulation resulting in p-mTOR (ser 2448) (McGonigle et al., 2002; Chiang and Abraham, 2005). mTOR also phosphorylates and regulates the S6 kinases by positively controlling protein translation (Nandagopal and Roux, 2015). The siRNA treatment of mTOR downregulated expression of mTOR and p70S6K proteins indicating the mTOR regulation (Wang et al., 2020). Hyperoxia exposure of mice for 72 h found diminished the expression of S6k proteins and inhibition of protein synthesis (Konsavage et al., 2010). This study found that Alda-1 pretreatment in mice prior to hyperoxia modulated both total mTOR and its various phosphorylated forms. In other studies pretreatment of Alda-1 ameliorated liver and cardiac function by inducing autophagy via Akt/mTOR pathway (Ge et al., 2011; Liu et al., 2020). Alda-1 may offer protection of HALI in mice by inducing autophagy via Akt/mTOR pathway.

The p-mTOR and phosphor p70S6 kinase (Thr 389) levels are increased in hyperoxia vs. normoxia because cells have the adaptive response to survive the apoptosis execution pathway as represented by cytochrome c release. The cell that survived during hyperoxia could be specific progenitor type cells or cells which are dedifferentiated (Kotton and Morrisey, 2014; Fukumoto et al., 2019). Certain cell types in the lung like club cells are resistant to apoptosis and resides in bronchioles, near to alveoli and basal lung cells and plays a crucial role in regeneration, repair and homeostasis of epithelial cells when damaged (Rock et al., 2010; Volckaert and De Langhe, 2014; Fukumoto et al., 2019).Cell types such as type 1 and type 2 alveolar epithelial cells undergo apoptosis during hyperoxia conditions (Fukumoto et al., 2019). Therefore, it is possible that lung homogenates show both increased survival signals and enhanced apoptotic signals under hyperoxia.

The p-Akt, mTOR, p-MTOR and p-p70s6kinase expressions were increased in lung endothelial cells exposed to hyperoxia for 24 h (Ahmad et al., 2006; Zhang et al., 2015). However, exposure of lung endothelial cells to 48 h of hyperoxia shows decline of Akt/mTOR pathway proteins (Ahmad et al., 2006). Probably the early activation of Akt could support and help in maintaining increased expression of p-Akt, mTOR associated proteins under 24 h of hyperoxia but not under 48 h (Ahmad et al., 2006). The pretreatment of Alda-1 may enhance the expression of these proteins in endothelial cells after hyperoxia as it enhanced Akt/mTOR protein levels. Previous reports combined with our data suggests that Alda-1 protects lung endothelial cells from oxidative stress induced cell death. Alda-1 activates mitochondrial Aldh2 and Aldh2 plays a pivotal role in Akt/mTOR activation to confer protection by inducing autophagy (Ge et al., 2011; Liu et al., 2020).There was no statistical significance in mTOR pathway results. Future studies will evaluate protein expression in lysates exposed to 24 h of hyperoxia with increased number of mice to analyze the statistics under 48 h of hyperoxia. Several other molecules activate mTOR and attenuate oxidative stress including: NV-5138, L-leucine, 3 BDO (3-benzyl-5-((2-nitrophenoxy) methyl)–dihydrofuran-2(3H)-one), and MHY1485 (Boultwood et al., 2013; Ge et al., 2014; Zhao et al., 2016; Sengupta et al., 2019).

These results demonstrate the cytoprotective role of Alda-1 in HALI. Activation of ALDH2 by Alda-1 restores ALDH2 activity and attenuates oxidative stress-induced injury in various animal models of atherosclerosis, Parkinson’s disease, ischemia/reperfusion injury, heart failure, and stroke (Chen et al., 2014; Fu et al., 2014; Breitzig et al., 2016; Lu et al., 2017; Zhang et al., 2018). Formulating a route to effectively administer Alda-1 in humans may protect against the lung damage caused by supplemental oxygen. Alda-1 pharmacokinetics have been extensively studied in rodent models (Chen et al., 2014). Given its non-toxicity in rodent models, these findings may easily be translated from bench to bedside for treatment of serious lung diseases and injuries. In conclusion, future studies aimed at understanding the therapeutic role of Alda-1 are vital for the treatment of ALI.

## Data Availability Statement

The raw data supporting the conclusions of this article will be made available by the authors, without undue reservation.

## Ethics Statement

The animal study was reviewed and approved by University of South Florida.

## Author Contributions

Concept and experimental design: SP, NK, LG, JF, RS, and MB. Experiments performed: SP, ML, HH-C, VN, and LG. Figure preparations and data analyzed: SP, JF, and SK. Manuscript revision and proof reading: NK, MA, LG, VN, RS, RL, MB, and HH-C. Final approval of the manuscript: LG and NK.

## Funding

LG is supported by the AHA National Scientist Development Grant 17SDG32780002 and NK is supported by the National Institutes of Health R01 HL105932.

## Conflict of Interest

The authors declare that the research was conducted in the absence of any commercial or financial relationships that could be construed as a potential conflict of interest.

## Abbreviations

NO, Normoxia; HO, Hyperoxia; ALI, Acute lung injury; HALI, Hyperoxia induced acute lung injury.
