# Supplementary figures and images for: Alda-1 Attenuates Hyperoxia-Induced Acute Lung Injury in Mice
Source: Front Pharmacol. 2021 Jan 8;11:597942. doi: 10.3389/fphar.2020.597942 (PMC7883597; doi:10.3389/fphar.2020.597942)

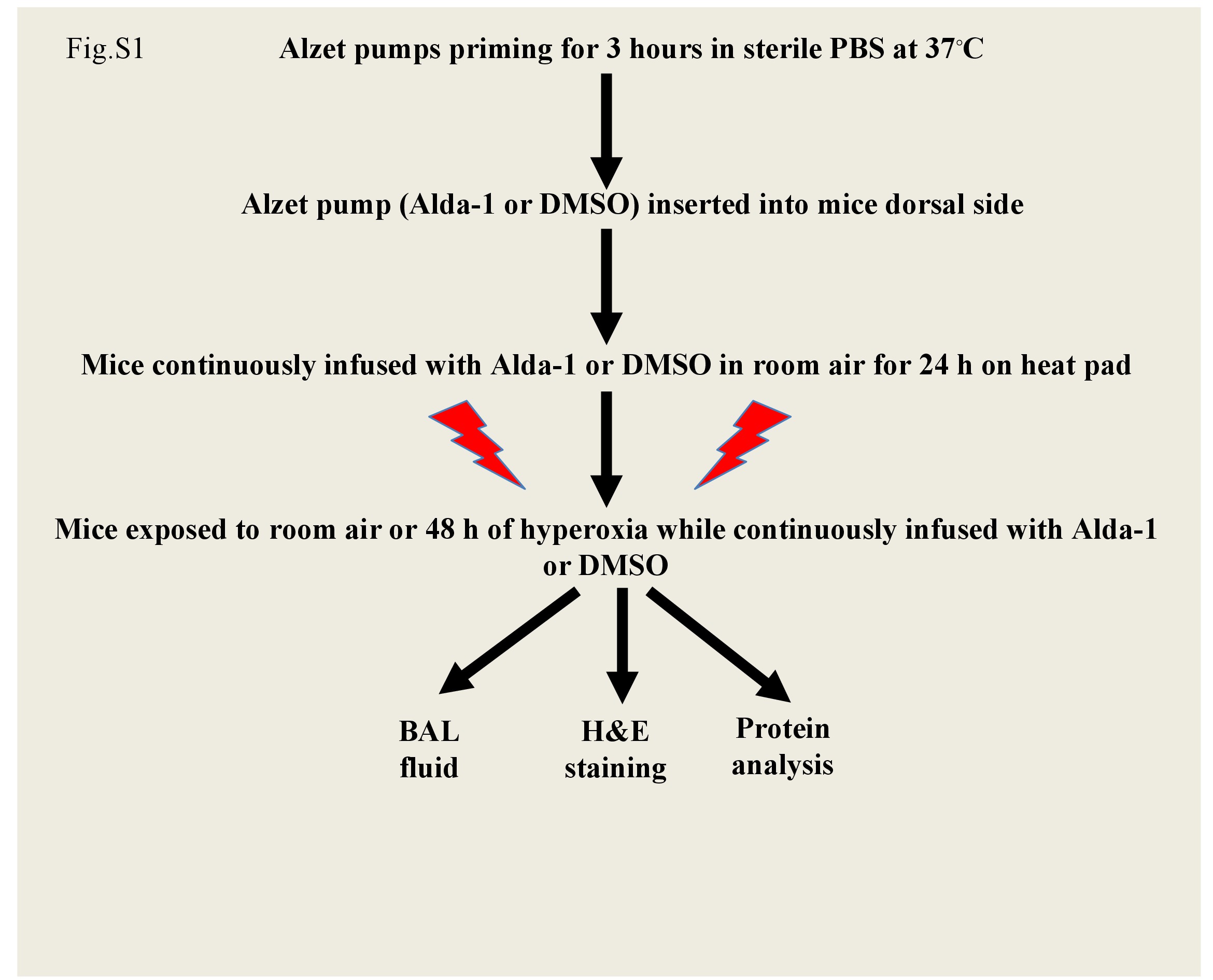

Supplement: Supplementary file 1 [file image1.jpeg]
